# Survey of Anti-Pathogen Antibody Levels in Myalgic Encephalomyelitis/Chronic Fatigue Syndrome

**DOI:** 10.3390/proteomes10020021

**Published:** 2022-06-13

**Authors:** Adam J. O’Neal, Katherine A. Glass, Christopher J. Emig, Adela A. Vitug, Steven J. Henry, Dikoma C. Shungu, Xiangling Mao, Susan M. Levine, Maureen R. Hanson

**Affiliations:** 1 Department of Molecular Biology and Genetics, Cornell University, Ithaca, NY 14853, USA; ajo39@cornell.edu (A.J.O.); kg432@cornell.edu (K.A.G.); cfssuelev@earthlink.net (S.M.L.); 2 Augmenta Bioworks, Inc., Menlo Park, CA 94025, USA; chris@augbio.com (C.J.E.); adela@augbio.com (A.A.V.); steve@augbio.com (S.J.H.); 3 Department of Radiology, Weill Cornell Medicine, New York, NY 10021, USA; dcs7001@med.cornell.edu (D.C.S.); xim2004@med.cornell.edu (X.M.)

**Keywords:** serology, herpesvirus, enterovirus, sex differences, ME/CFS, plasma, immune response

## Abstract

Infectious pathogens are implicated in the etiology of myalgic encephalomyelitis/chronic fatigue syndrome (ME/CFS) because of the occurrence of outbreaks of the disease. While a number of different infectious agents have been associated with the onset of ME/CFS, the identity of a specific organism has been difficult to determine in individual cases. The aim of our study is to survey ME/CFS subjects for evidence of an infectious trigger and/or evidence of immune dysregulation via serological testing of plasma samples for antibodies to 122 different pathogen antigens. Immune profiles were compared to age-, sex-, and BMI-matched controls to provide a basis for comparison. Antibody levels to individual antigens surveyed in this study do not implicate any one of the pathogens in ME/CFS, nor do they rule out common pathogens that frequently infect the US population. However, our results revealed sex-based differences in steady-state humoral immunity, both within the ME/CFS cohort and when compared to trends seen in the healthy control cohort.

## 1. Introduction

Myalgic encephalomyelitis/chronic fatigue syndrome (ME/CFS) is a complex, multi-system disease; its diagnosis requires the occurrence of profound fatigue, post-exertional malaise, sleep dysfunction, pain, two or more cognitive/neurological manifestations, and at least one symptom related to autonomic dysfunction, neuroendocrine dysfunction, or immune dysfunction, according to the Canadian Consensus Criteria (CCC) [1]. In the acute phase of illness, many ME/CFS sufferers complain of a flu-like illness characterized by fever, chills, sore throat, headache, and muscle aches. Acute illnesses prior to long-term chronic illness have been observed in both sporadic, isolated cases of ME/CFS as well as in clusters and outbreaks, where tens or hundreds of individuals are affected over a short period of time in the same general geographic location [2].

Reports of epidemic events with symptom constellations reminiscent of ME/CFS have been recorded as early as the late 1600s to the mid-1700s [2,3,4]. Since these initial outbreaks, an infectious culprit in ME/CFS disease onset has been suspected. Early investigations following the 1934 Los Angeles County Hospital outbreak [5] focused on enteroviruses (EVs) as disease initiators due to: (1) the spatiotemporal overlap between ME/CFS outbreaks and known poliomyelitis epidemics of the time; (2) the seasonality of ME/CFS outbreaks matching those of enteroviral outbreaks; and (3) the ME/CFS symptom constellation overlapping with symptoms described across known chronic enteroviral clinical outcomes [6,7,8]. Although many clues point to enteroviruses as etiologic agents of this disease, other research groups have put forward additional causal agents as potential disease initiators in ME/CFS—including, but not limited to, Brucella, *Chlamydia pneumoniae*, *Coxiella burnetti*, cytomegalovirus, Epstein-Barr virus, other human herpesviruses, hepatitis C virus, human lentiviruses, human T-cell leukemia virus II-like virus, parvovirus B19, Borna virus, spumavirus, and *Toxoplasma gondii* [6,9,10,11,12,13].

Evidence for immune dysfunction in some ME/CFS sufferers includes reduced natural killer cell toxicity, altered inflammatory cytokine and immunoglobulin profiles, inconsistent reports on altered T- and B-cell function, and an increased incidence and family history of other immune disorders and autoimmune disorders such as fibromyalgia and Hashimoto’s thyroiditis [14,15]. To explore whether evidence exists for an infectious trigger and/or immune dysregulation in ME/CFS, we surveyed plasma samples from ME/CFS subjects and matched controls for antibodies to 122 different pathogenic antigens. The aim of our study is to determine whether individuals with ME/CFS exhibit higher levels of antibodies to a pathogen in comparison to controls and/or evidence of an altered immune system based on anti-pathogen antibody profiles. While absence of historical exposure and antibodies to a rare pathogen would provide evidence against that pathogen as causal in ME/CFS, our assays provide no information regarding the possibility that a pathogen family that frequently circulates amidst the general population might result in ME/CFS in a subset of those infected.

## 2. Materials and Methods

### 2.1. ME/CFS Case Selection and Sample Acquisition

ME/CFS cases and healthy controls were identified by Geoffrey Moore, M.D. (Ithaca, NY, USA), John Chia, M.D. (Los Angeles, CA, USA), and Susan Levine, M.D. (Manhattan, NY, USA) between 15 October 2015 and 6 March 2020. A total of 59 ME/CFS cases and 44 healthy controls were included in this case–control cross-sectional study. Individuals were diagnosed with ME/CFS if they met the Canadian Consensus Criteria for ME/CFS [1] and controls were eligible if they were healthy, had not been diagnosed with depression, were sedentary, were between 18 and 70 years old, were non-smokers, were not pregnant or breast feeding, were not diabetic, and did not display a metabolic, cardiovascular, and/or other neuroimmune disease. Patients included in the study did not report the use of immune-modulating drugs.

Peripheral blood from an antecubital vein was drawn into EDTA tubes. Once collected, blood tubes were put on ice and taken to labs for immediate separation of plasma, which was stored on the same day of collection at −80 °C until further use. Participants’ age, sex, and age of onset of ME/CFS were recorded. The Bell Disability Scale [16] and Short Form-36 Health Survey [17] were administered to each participant on the day of blood sample collection. Written consent was obtained from all participants, and all protocols were approved by Weill Cornell Medical College, Protocol # 1708018518, Ithaca College IRB # 1017-12Dx2.

### 2.2. Augmenta Serological Testing

A panel of Luminex xMAP beads was constructed by coupling beads to recombinant proteins, inactivated viruses, and inactivated cell cultures (Table S1). Native antigens were inactivated by one or more standard methodologies (heat treatment, UV irradiation, chemical inactivation, gamma irradiation, detergent, or electron beam irradiation) to render them safer for handling in BSL-2 conditions. Most antigens were received live from the ATCC. All antigens were buffer-exchanged into phosphate-buffered saline (PBS) with size exclusion chromatography prior to coupling. Concentrations were measured using the Pierce Rapid Gold BCA protein assay before and after buffer exchange.

Each antigen was assigned a unique Luminex bead region. The corresponding bead stock was resuspended and coupled to purified antigen via standard sulfo-NHS/EDC linking chemistry. Reactions were quenched, and the beads were washed and blocked against nonspecific binding with Surmodics StabilGuard. Up to 24 conjugations were performed in parallel using a Hamilton Liquid Handler (Hamilton Company, Reno, NV, USA). Conjugated beads were pooled into three separate panels for the assay of subject samples.

Three quality control measurements were run on every bead–antigen conjugation lot. First, the final bead concentration was determined using a BD AccuriC6 flow cytometer (BD, Franklin Lakes, NJ, USA) to compare the conjugated beads to bead standards of known size and concentration. Second, the NanoOrange Protein Quantitation assay was used to fluorometrically detect the presence of protein on coupled beads relative to uncoupled beads. Third, a full-scale Luminex assay was performed using intravenous immunoglobulin (IVIg) from a broad spectrum of donors to confirm positive signal detection (i.e., antibody binding to antigen-coupled beads above background). 

For all three panels, the study samples were run in triplicate at 1:500, 1:1000, and 1:2000 dilutions. For ease of analysis, only data from the 1:1000 dilution was used. Two replicates of CONSV3 control serum (Sigma, St. Louis, MO, USA) and three replicates of a single control plasma were used as technical controls in all plates, and three replicates of a secondary-only negative control (blank) were also included in all plates. All sample wells had bovine serum albumin (BSA) beads as a negative control and a bead conjugated to PE anti-human CD38/PE as a positive instrument control. The mixture was washed twice before the addition of a phycoerythrin (PE)-conjugated Goat Anti-Human IgM+IgG+IgA reporter antibody. Samples were washed twice and read on a Luminex 200 flow cytometer (Luminex, Austin, TX, USA) within 2 h of the final wash. The log2 difference in median fluorescence intensity (MFI) between a given antigen-coupled bead lot and its internal batch-specific negative control (BSA) was computed. Triplicates were averaged. Normalization was performed to compensate for technical variations between plates by centering the mean global intensity of a reference sample on each plate. MFI data were log2-transformed prior to statistical analyses. Each antigen’s log2MFI value was calculated by subtracting the MFI value from a PBS negative control.

### 2.3. Statistical Analysis

All data were processed and analyzed using R version 3.5.2 (21 February 2019) via RStudio Version 1.4.1717 (RStudio, Boston, MA, USA) and/or MetaboAnalyst version 5.0 (https://www.metaboanalyst.ca, accessed on 21 February 2019). The outlier amended dataset was created by first identifying outliers using the 1.5*IQR rule and then replacing outliers using the Bayesian PCA (BPCA) estimation method for outlier replacement. The Wilcoxon–Mann–Whitney U-test was used to determine the significance of differences (*p* < 0.05) in ME/CFS vs. control subjects across all sample population characteristic measurements (Table 1) and for all individual pathogenic antigens (Table S1). For the 122 antigens, correction for multiple comparisons was done via the Benjamini–Hochberg method for false discovery rate (FDR) correction. We report for each antigen both the *p*-value (α = 0.05) and q-value (α = 0.05) to provide clarity about the level of statistical significance.

Principal component analysis (PCA) was used to reduce the dimensionality of the anti-pathogen antibody data. The data correlation matrix was then calculated, and eigenvalue decomposition on the matrix was performed. Cluster tendency of the datasets was assessed using the Hopkins statistic (H) [18] by measuring the probability that a given data set is uniformly randomly distributed [19]. A H-value close to 1 indicates the data is highly clustered, and random data will result in values close to or below 0.5. PCA; cluster tendency was performed using the R packages ggplot2, factoextra, and clustertend. Fold change and volcano plot analyses were completed using MetaboAnalyst version 5.0 with a fold-change threshold of 1.01 and a *p*-value of 0.05.

## 3. Results

### 3.1. Subject Characteristics

In total, the study population consisted of 105 subjects, including 44 healthy control subjects and 59 ME/CFS subjects (Table 1). The healthy control cohort consisted of 29 females and 15 males, while the ME/CFS cohort consisted of 47 females and 12 males (Table 1). All individuals who were selected met the Canadian Consensus Criteria for ME/CFS. The average age was similar between groups at 46.2 ± 10.8 years in ME/CFS subjects and 42.1 ± 14.2 years in controls (*p* = 0.11, Table 1). Average body mass index (BMI) was also similar between groups at 26.5 ± 5.8 in ME/CFS and 27.5 ± 5.0 in controls (*p* = 0.30, Table 1); 43% of ME/CFS subjects indicated a gradual onset of disease, while 57% described a sudden onset of disease (Table 1). ME/CFS illness duration varied, with a range of 1 to 38 years and an average of 12.1 ± 9.6 years (Table 1). ME/CFS onset occurred in all subjects before SARS-COV2 emerged. Bell Scale ratings were significantly different between groups, with scores averaging 34.0 ± 12.4 and 95.5 ± 8.4 for ME/CFS subjects and controls, respectively (*p* < 0.001, Table 1). Both the physical and mental component scores (PCS and MCS, respectively) derived from the SF-36 short survey were, as expected, higher in the control group (*p* < 0.001, Table 1), indicating better health. No subjects were reported to be taking immune-modulating drugs.

### 3.2. Anti-Pathogen Antibody Profiles between ME/CFS Cases and Controls

To determine whether differences in anti-pathogen antibody levels exist between the ME/CFS and control cohorts, we (1) used PCA to see if broad trends or differences could be identified when comparing serological responses to all 122 antigens collectively and (2) compared the serological responses to each antigen. Individual antigen differences between ME/CFS and control subjects were explored to determine whether they might implicate an etiological agent or ME/CFS disease perpetuator. We first compared all ME/CFS cases and controls and then looked at each sex independently. 

#### 3.2.1. Dataset Amendment for Outlier Influence Does Not Significantly Alter Findings Related to Antibody Profile Differences

**Figure 1:**
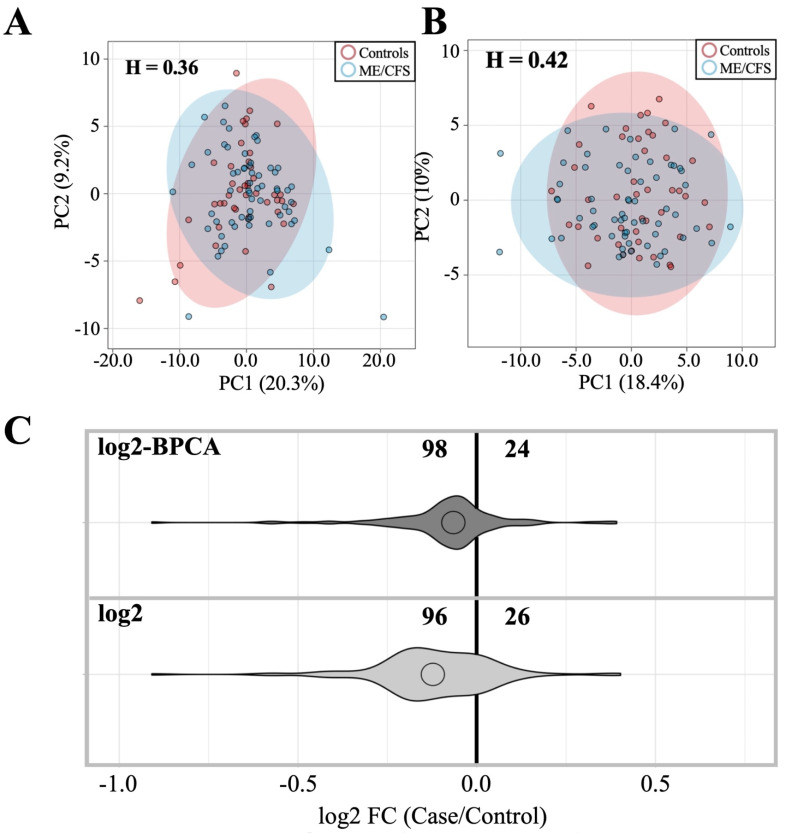
Comparison of ME/CFS and control antibody profiles with and without outlier replacement. (**A**) PCA of ME/CFS vs. control subjects using log2 dataset. (**B**) PCA of ME/CFS vs. control using log2 BPCA-estimated outlier replacement dataset. H = Hopkins statistic. PCA legend indicates patients (blue) vs. controls (red). (**C**) Violin plot depicting fold-change relationship between case and control subjects within each dataset.

Extensive overlap in case (*n* = 59) and control (*n* = 44) ellipses, proximity of case and control population means, as well as Hopkins statistic scores less than 0.5 indicate that ME/CFS and healthy control cohorts are indistinguishable in terms of antibody levels to the 122 antigens surveyed when the antigens are considered collectively via PCA (Figure 1A,B). Roughly 80% of the antigens (96–98/122) trend toward decreased mean antibody levels in cases relative to controls, and this trend is maintained when effects of outlier influence are attenuated (Figure 1C, Tables S2 and S3). Outlier influence attenuation was carried out to ensure trends in the data were representative of the entire cohort instead of artifactual trends influenced by one or a few subjects with dramatic differences in log2MFI values, possibly due to recent infection or an unusual absence of exposure to a common pathogen. 

**Figure 2:**
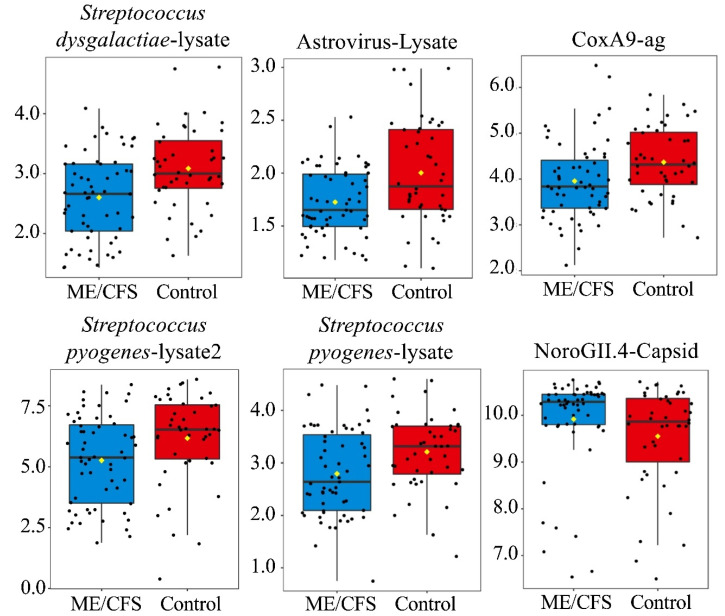
Boxplots-6 antigens with significantly different antibody levels from controls following outlier replacement (*p* < 0.05, q > 0.05). Antigens are listed from left to right and top to bottom based on *p*-value (Table 2). Y-axis = log2MFI values. Yellow diamond indicates sample mean. Black line running horizontal through the boxplot indicates sample median.

Table S1 explains antigen nomenclature. In total, seven antigens (Astrovirus, Coxsackievirus A9, Norovirus GII.4 capsid protein (VP1), Rhinovirus A15, *Streptococcus pyogenes* 1, and *Streptococcus pyogenes* 2, and *Streptococcus dysgalactiae*) are shown to have significantly different antibody levels between cases and controls (*p* < 0.05, q > 0.05), with the significance of RhinoA15-lysate being limited to the non-outlier amended dataset (Table 2, Figure 2, Tables S2 and S3). However, for the total population, none of these seven antigens remained significant after FDR correction. Of the six significant antigens following BPCA replacement, Norovirus GII.4-VP1 was the only antigen with increased antibody levels in cases relative to controls (Table 2, Figure 2, Tables S2 and S3). The remaining analyses presented herein were conducted on the outlier amended dataset unless otherwise stated. 

#### 3.2.2. Sex-Based Subgrouping Reveals Differences between Male and Female Antibody Profiles

**Figure 3:**
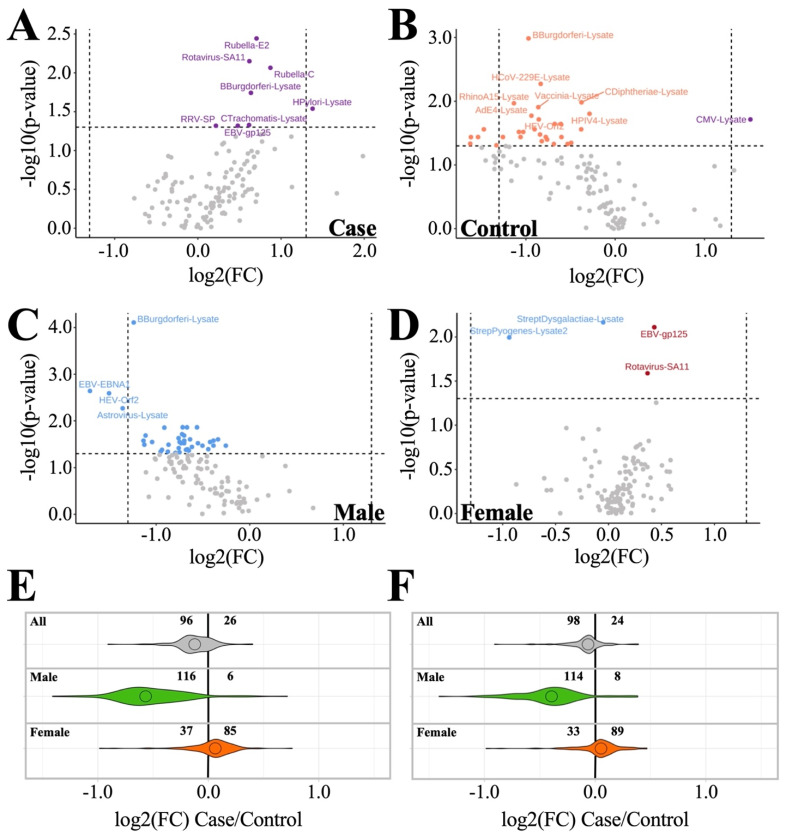
Inter- and intra-cohort sex-specific antibody profile trends. (**A**,**B**) Intra-cohort comparisons (female/male). (**A**) ME/CFS male vs. ME/CFS female. (**B**) Control male vs. control female. (**C**,**D**) Inter-cohort comparisons (case/control). (**C**) ME/CFS male vs. control male. (**D**) ME/CFS female vs. control female. (Purple) antigens with average antibody levels higher in females. (Orange) antigens with average antibody levels higher in males. (Blue) antigens with average antibody levels lower in cases than controls. (Red) antigens with average antibody levels lower in controls than in cases. Horizontal line in volcano plot indicates *p* = 0.05. (**E**,**F**) Violin plots depicting fold change. All, male, and female subgroup antigen fold changes between case and control are shown. Less than zero indicates lower in case vs. control. (**E**) Log2 dataset without outlier replacement; (**F**) log2 dataset with BPCA-estimated outlier replacement.

When the dataset is subgrouped according to male (*n* = 27) and female (*n* = 76) within both case and control cohorts, we discover that sex-specific antibody profile differences occur both within and between cohorts (Figure 3, Table 3 and Table S4). Intra-cohort analysis within the ME/CFS cohort (Figure 3A) revealed that males show a trend toward lower mean antibody levels compared to females for most antigens tested. Of these antigens, eight antibody levels were shown to be significantly different (*p* < 0.05) between ME/CFS male and female subjects (Rubella-E2, Rotavirus-SA11, Rubella-C, *B. burgdorferi*-lysate, H. pylori-lysate, RRV-SP, *C. Trachomatis*, and EBV-gp125) (Figure 3A). Conversely, the control cohort showed males as having higher mean antibody levels than females for most antigens tested, with most antigens having log2 fold changes (female/male) less than zero (Figure 3B). Of these 13 antigens, CMV-lysate was the only one with an average antibody level higher in control females rather than lower (Figure 3B). 

When looking at inter-cohort analyses (Figure 3C–F), we see male subjects (*n* = 27) follow the same general trends as the total subject dataset (Figure 3A,C,E) but with even greater contrast between patients and controls, as indicated by (1) an increase in the number of antigens (114/116, up from 96/98 out of 122), showing a trend toward decreased antibody levels in cases vs. controls, and (2) an increase in the number of antigen levels found to be significantly different (*p* < 0.05) between patient and control (42, up from 6/7 out of 122) (Figure 3C,E,F, Table 3 and Table S4). Of these, *B. burgdorferi*-lysate is the only antigen with antibody levels found to be significantly different following false discovery rate (FDR) correction (q < 0.05) (Figure 3C). Females (*n* = 76) show an opposite trend when compared to the total and male subject datasets (Figure 3A,E,F, Table 3). ME/CFS females have only 33–37/122 antigens with antibody levels trending lower than control females, while most antigens (85–89/122) exhibit increased antibody levels in ME/CFS cases relative to control (Figure 3D–F). Four antigens show significant differences at *p* < 0.05, but zero antigens are identified as significant following FDR correction. In short, males and females show opposing relationships of antibody levels when comparing ME/CFS and control subjects. 

Of the anti-pathogen antibody levels found to have *p* < 0.05 differences between patients and controls in the male and female cohorts, Rotavirus-SA11 is the only antigen with significantly different antibody levels in both cohorts. The two cohorts have opposing Rotavirus SA-11 findings, with ME/CFS males having lower antibody levels than control males and ME/CFS females having higher antibody levels than control females (Table 3 and Table S4). 

#### 3.2.3. Age and Illness Duration Subgroup Analyses Reveals Additional Insights into Antibody Profiles 

**Figure 4:**
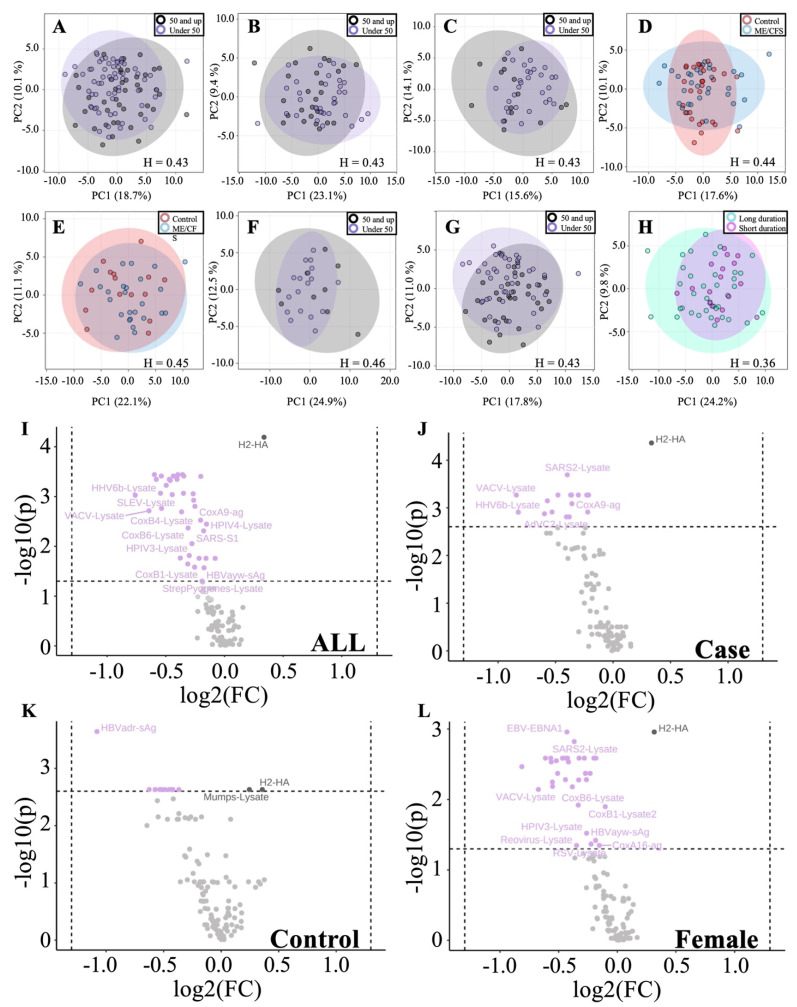
Age- and illness-duration-based antibody profile comparisons. (**A**) All subjects under 50 vs. all subjects 50 and up. (**B**) ME/CFS under 50 vs. ME/CFS 50 and up. (**C**) Control under 50 vs. control over 50. (**D**) ME/CFS vs. control under 50. (**E**) ME/CFS vs. control over 50. (**F**) Male under 50 vs. male over 50. (**G**) Female under 50 vs. female over 50. (**H**) ME/CFS short (≤5 years) vs. long (>5 years) illness duration. H = Hopkins statistic. Hopkins statistic is individually given for each PCA. (Black) 50 and up. (Purple) under 50. (Red) control. (Blue) ME/CFS. (Turquoise) long duration. (Fuchsia) short duration. (**I**–**L**) Volcano plot highlighting antigens with significantly different levels between age groups overall and within experimental subgroups (Over 50/Under 50). (**I**) All subjects under 50 vs. all subjects over 50. (**J**) ME/CFS under 50 vs. ME/CFS over 50. (**K**) Control under 50 vs. control over 50. (**L**) Female under 50 vs. female over 50. (Light purple) antigen level is lower in the over 50 age group. (Black) antigens higher in the over 50 age group.

ME/CFS and control cohorts were organized by subgroups of under 50 (*n* = 62) vs. over 50 (*n* = 41) and compared both within and between groups (Figure 4A–E). Hopkins statistics scores for all five comparisons fall below 0.5 and, therefore, suggest we are unable to distinguish between the subgroups compared. In addition, we compared sex-based age differences, including comparing females under 50 to females over 50 (Figure 4F) as well as males under 50 to males over 50 (Figure 4G). Once again, Hopkins statistics scores indicate the two populations are not distinguishable based on antibody responses to the 122 pathogen antigens surveyed. Finally, subjects were stratified based on short- (less than or equal to 5 years) vs. long-term (greater than 5 years) illness duration, and PCA again indicated there were no significant differences (Figure 4H). 

Statistical analysis of individual antigens shows a subset of antibody responses to specific antigens significantly differ (*p* < 0.05, q < 0.05) between under 50 and over 50 age groups when considering all subjects (Figure 4I), ME/CFS subjects alone (Figure 4J), control subjects alone (Figure 4K), and female subjects alone (Figure 4L). A complete list of identified pathogens within each grouping is presented in Table S5.

## 4. Discussion

Pathogens have been continuously put forward as potential ME/CFS disease initiators and/or exacerbating agents. The occurrence of clustered outbreaks, the often sudden onset of disease, and reports of “flu-like” symptoms during acute phases of disease progression suggest an infectious pathogenic agent. Survey data obtained from ME/CFS subjects enrolled in this study is consistent with this theory, as 33 of 58 survey respondents with ME/CFS reported a sudden disease onset, with 17 of the 33 respondents reporting a viral or viral-like disease at onset, characterized by sore throat, low-grade fever, etc.

Our study population consisted of ME/CFS and healthy control subjects matched for age, sex, and BMI (Table 1). In total, we surveyed the antibody profile of 59 ME/CFS and 44 healthy control subjects to 122 pathogenic antigens. Antigens surveyed were chosen to represent common human pathogens across all seven Baltimore classification viral types as well as subsets of bacterial and protozoan clades (Table S1). The antibody detection method employed in our study is unable to discriminate between antibody classes because the secondary antibody that was used detects a combination of IgG, IgA, and IgM. Because this assay has not been cross-validated with established diagnostic ELISAs that are usually Ig class-specific, our knowledge of the connection between the statistical significance of the antibody levels measured here and clinical significance is limited. However, the Luminex platform is a well-established epidemiological and basic science research tool for multiplex serology, and antibody responses measured via Luminex typically correlate well with diagnostic ELISAs [20,21,22]. Insight into ME/CFS anti-pathogen immune profiles reveals support for sex- and age-based immune differences that may exist across ME/CFS disease demographics.

Sex-based subgrouping shows that male and female cases differ in their anti-pathogen immune profiles. Female subjects had 85–89/122 antigens with log2 fold changes greater than zero in cases relative to controls, whereas 114–116/122 antigens in males showed log2 fold changes below zero (Figure 3C–F). While most of the differences were not significant, the opposite trends between male and female antibody levels indicate it is inappropriate to combine data from the two sexes. Immunological differences between sexes are well known; for example, susceptibility to various autoimmune diseases varies between sexes [23]. Sex differences exist in ME/CFS, given that more women than men are diagnosed with the disease [24]. Furthermore, plasma metabolites differ between male and female ME/CFS cases and controls [25]. The contrast between males and females is even further magnified when we realize the only shared significantly different antibody level between sexes is Rotavirus-SA11 (*p* < 0.05, q > 0.05), whose correlation with controls is opposite between males and females. 

Rotaviruses typically infect infants and children with no significant sex-specific difference in burden between males and females. Adult infections are rare but still occur. Typically, adult infection is derived from endemic disease, epidemic outbreaks, travel-related gastroenteritis, and infections transmitted from children to adults [26]. Of the adult outbreaks, no preference is seen between male or female adults [27]. Lack of congruency between the epidemiology of ME/CFS and rotavirus suggests that the finding of rotavirus significance is not related to ME/CFS disease initiation or progression. Factors leading to this conclusion include contrasting epidemic seasonality between ME/CFS and rotavirus epidemics, small symptom overlap, and deviations when comparing expected incidence, distribution, and demographics of afflicted individuals. 

Overall, females exhibit levels of antibodies to four antigens that are significantly different (*p* < 0.05, q > 0.05) between case and control, with antibody levels to two antigens (*Streptococcus dysgalactiae*-lysate and *Streptococcus pyogenes*-lysate) being decreased in case vs. control and two antigens (EBV-gp125 and Rotavirus SA11) being increased in case vs. control (Figure 3D). The finding of increased EBV antibodies to viral capsid antigen (VCA) glycoprotein 125 (EBV-gp125, encoded by the BALF4 gene) in our female cohort suggests a possible link to EBV (Table S2). However, the difference was not significant after FDR correction. Additionally, we found that, altogether, females had higher antibody responses to EBV-gp125 than males (*p* < 0.05, q > 0.05), which supports the conclusion that this result is specific to females and not a statistical artifact of the smaller sample size for males in this study. We also measured antibody levels to one other EBV antigen, Epstein-Barr nuclear antigen 1 (EBNA-1), and did not find any difference between case and control females. This is not surprising, as approximately 90% of adults worldwide have been infected with EBV and anti-EBNA-1 IgG may persist for life, simply indicating a past infection [28]. Differences in EBV humoral immunity between ME/CFS patients and healthy controls have been found in other studies, including several that also used the Canadian Consensus Criteria to select ME/CFS subjects [22,29,30]. Enhanced IgG levels against EBNA-6 were found using a peptide microarray platform [29], but the same result was not significant via Luminex [22].

Clinical diagnostic assays for EBV for either recent or past infection use a different viral capsid antigen, p18 [31], so EBV-gp125 antibody responses have not been as widely studied. EBV-gp125 has been examined in one other serology study with ME/CFS patients, but that study did not find differences between patients and controls when measuring IgG and IgM separately and looking at both sexes collectively [22]. In our assay, IgM and IgA against EBV-gp125 are also contributing to the signal. These isotypes are associated with recent infection or reactivation [28]. Unfortunately, we cannot conclude whether or not our result is clinically significant without confirming the result via ELISA and assaying additional EBV antigens, including those present in already developed diagnostic ELISAs for EBV. Additionally, the assessment of EBV reactivation is outside the scope of our assay. Although they did not subset their study population into males and females, Domingues et al. found a negative association between controls and patients that did not self-identify as having an infectious disease trigger for seropositivity to the EBV antigens VCA (p18) and EBNA-1 [30]. The result was similar when adjusting for sex as a confounding factor. In the same group of subjects, but without evaluating any subsets, the levels of anti-VCA IgG and seropositivity status were not different between ME/CFS subjects and controls [32]. While our broad survey of a variety of pathogens did not thoroughly investigate the EBV antibody response, this finding of higher antibody responses to EBV-gp125 in female patients warrants further investigation and confirmation with a larger sample size. 

Males have a surprising set of 42 antibody levels that are significantly lower (*p* < 0.05) compared to controls, with *B. burgderfori*-lysate being the only antigen significant after FDR correction (q < 0.05) (Figure 3C, Table 3). *B. burgdorferi*-lysate is derived from the Lyme disease spirochete, a tick-borne pathogen that, in some individuals, is thought to cause chronic Lyme disease, which has symptoms that overlap with ME/CFS. Results obtained from our male cohort do not implicate Lyme disease as a possible disease culprit because the average case antibody level for this antigen is lower than control. One caveat to this result is that this antigen is derived from whole lysate, which has a higher incidence of nonspecific binding than recombinant antigens. Follow-up studies focused on antibody responses in ME/CFS and/or chronic Lyme disease using recombinant protein antigens would help confirm or refute this finding. Despite a relatively small male sample size, we found more differences in antibody levels between ME/CFS subjects and controls in males than in females. One hypothesis, put forward by Domingues et al. to explain immunological findings regarding human herpesviruses in ME/CFS, is that CD4+ regulatory T-cells are hyperactive, resulting in the suppression of humoral immune responses [33]. Dysregulation of immune cells in males may thus result in the observed lower levels of antibodies not only to *B. burgdorferi* but also to a large number of pathogens (Figure 3). 

Age-based cohort stratification provides additional insight into humoral immune biology, with a large number of antigens identified with significantly different antibody levels between subjects under 50 and those over 50 (Figure 4A–C,G,I–L). Age 50 is a common age for menopause in females. Although PCA does not separate the general subgroups, comparing all subjects under 50 to all subjects over 50 (Figure 4I), as well as females under 50 to females over 50 (Figure 4L), we see that roughly 1/3 of antigens are significantly lower in the 50-and-over age group following FDR correction. This trend is maintained when stratifying by age within the case-only cohort (Figure 4J), but to a lesser degree, with fewer identified antigens.

Of the 122 antigens for which antibodies were assayed, 49 represent positive sense single-stranded RNA viruses. Of these 49, the majority are members of the Picornaviridae, which includes enteroviruses. Non-polio enteroviruses have been implicated in outbreaks of ME/CFS. In our study, antibodies to 15 coxsackievirus antigens, 1 EV71 antigen, 1 EV68 antigen, 6 poliovirus antigens, and 3 rhinovirus antigens—representing five groups of enteroviruses—were assayed (Table S1). Of these, only the levels of Coxsackie virus A9 were significantly different (*p* < 0.05, q > 0.05) between male cases and male controls, with lower levels in cases (Figure 2, Table S3). The current study is not able to determine whether or not an enterovirus might have incited a large proportion of ME/CFS cases because enteroviral infections occur in the general population worldwide so often each year [34]. In the US alone, there are 30–50 million enteroviral infections each year, of which 10–15 million are symptomatic [35]. As a result, even if enteroviruses were inciting agents of most ME/CFS cases, both cases and controls would be expected to have largely overlapping levels of antibodies. Our dataset shows antibodies to the enterovirus antigens surveyed exhibit very small fold-change differences between cohorts, which is expected due to the aforementioned frequency of infections. Our assays cannot determine whether some subjects have been exposed to particular variants of certain enteroviruses that have not infected other subjects. The possibility remains that ME/CFS cases arise from an uncommon variant of one or more enteroviruses or another type of virus and/or an uncommon reaction to a common endemic virus.

One limitation of this study is that the antibody response we measured is a combination of IgG, IgA, and IgM antibodies. We recommend that future studies in this area investigate multiple Ig classes and subclasses independently. If a pathogen that commonly infects a large percentage of the population (such as an enterovirus or a herpesvirus) is involved in ME/CFS etiology, it is likely that the differential humoral immune response to that pathogen could be highly nuanced and is simply not detected in our assay. For example, the presence of IgM and IgA against EBV VCA antigens or IgA and IgG against early antigen D are indicators of EBV reactivation that were not assayed in our study [28].

Another limitation of this study is that we did not attempt to determine who was seropositive and who was seronegative. Even though we subtracted the background signal, it is possible that some of the signals we detected are due to cross-reactivity or nonspecific binding, particularly to the antigens that are whole pathogen lysate rather than specific recombinant proteins. In this exploratory study, we wanted to consider all the data for as many pathogens and antigens as possible and to look for pathogens that warrant further follow-up studies. If any pathogens had stood out as potential causative agents of ME/CFS, we would have been interested in following up with confirmatory ELISAs, which include positive and negative control reference serums to help determine seropositivity status, in addition to measuring the relative antibody levels. Such assays could be done in future work, particularly in studies focused on a smaller number of pathogens, such as EBV and/or *B. burgdorferi.*

## 5. Conclusions

While examining levels of antibodies to whole proteins and lysates would not be expected to identify a disease-causing pathogen specific to ME/CFS if the pathogen routinely affects large numbers of individuals, rare pathogens could be implicated if they are found to be more prevalent in the case group. Although some rare pathogens were probed in our study, none show significant differences between cases and controls after false discovery rate correction. To the best of our knowledge, this is the first time that antibody levels to many of the pathogens investigated here have been explored in ME/CFS patients, including tick-borne encephalitis virus, hepatitis E virus, and human metapneumovirus. We also probed the largest number of adenoviruses (14) and enteroviruses (20, in five different groups) of any study to date. While the primary conclusion from our study is the absence of differences between antibody levels of patients and controls, this type of exploratory analysis using high-throughput, multiplex technologies is important to characterize the humoral immune response of ME/CFS patients and to continue the search for a possible infectious agent that triggers ME/CFS. We also show a trend in male antibody levels suggesting immunosuppression as well as differences in antibody levels with age. The subtle serological alterations between healthy controls and ME/CFS subjects found here should be interpreted cautiously, but these findings do contribute to the growing body of evidence that the immune system of ME/CFS patients is dysregulated [32,36]. We also recommend that future serological studies in ME/CFS patients should not combine males and females for analysis and that males should be studied in addition to females despite being a smaller subset of the patient population.

## Figures and Tables

**Table 1:** Study population characteristics. ^a^ PCS, physical component score; MCS, mental component score.

	ME/CFS	Controls	Mann–Whitney U-Test
Age	46.1 ± 10.5	42.1 ± 14.2	*p* = 0.11
Gender			
Female	47	29	NA
Male	12	15	NA
BMI (kg/m^2^)	26.5 ± 5.7	27.5 ± 5.0	*p* = 0.30
Ethnicity			
Hispanic or Latino	9	6	NA
Not Hispanic or Latino	46	37	NA
Unknown	4	1	NA
Race			
American Indian or Alaska Native	0	1	NA
Asian	2	5	NA
Black or African American	0	3	NA
Native Hawaiian or other Pacific Islander	1	0	NA
White	51	32	NA
Unknown	5	3	NA
Onset of disease			
Gradual	44%	NA	NA
Sudden	56%	NA	NA
Illness Duration (years)	12.1 ± 9.6	NA	NA
Bell Score	34.6 ± 12.2	95.5 ± 8.5	*p* < 0.001
SF-36			
Physical function	38.6 ± 19.3	94.2 ± 9.3	*p* < 0.001
Role physical	16.1 ± 18.8	98.3 ± 4.5	*p* < 0.001
Pain	41.9 ± 22.0	83.9 ± 16.7	*p* < 0.001
General health	22.9 ± 11.6	81.2 ± 13.9	*p* < 0.001
Vitality	17.6 ± 14.0	70.3 ± 17.8	*p* < 0.001
PCS ^a^	27.2 ± 7.2	56.1 ± 4.7	*p* < 0.001
Social function	26.3 ± 20.7	97.7 ± 11.5	*p* < 0.001
Role emotional	81.5 ± 24.8	97.0 ± 7.9	*p* < 0.001
Mental health	66.4 ± 18.8	83.9 ± 10.1	*p* < 0.001
MCS ^a^	45.1 ± 9.5	55.3 ± 5.1	*p* < 0.001

**Table 2:** Antigens with significantly different antibody levels between case and control with and without outlier replacement. Log2 MFI mean ± standard deviation and Mann–Whitney *p*-value are indicated for each pathogen. Data are shown for the original dataset as well as the outlier amended dataset. Blue indicates a lower antibody level in patients relative to controls, and red indicates a lower antibody level in controls relative to patients. *p* = *p*-value, q = q-value.

log2 Dataset					log2-BPCA Dataset				
Pathogen	ME/CFS	Control	*p*	q	Pathogen	ME/CFS	Control	*p*	q
*Streptococcus dysgalactiae*-lysate	2.7 ± 0.9	3.1 ± 0.9	0.003	0.39	*Streptococcus dysgalactiae*-lysate	2.6 ± 0.7	3.1 ± 0.7	0.001	0.18
*Streptococcus pyogenes*-lysate2	5.3 ± 1.8	6.2 ± 1.9	0.008	0.49	Astrovirus-Lysate	1.7 ± 0.3	2.0 ± 0.5	0.003	0.18
*Streptococcus pyogenes*-lysate	2.8 ± 0.8	3.1 ± 0.9	0.016	0.59	CoxA9	4.0 ± 0.8	4.4 ± 0.8	0.007	0.20
CoxA9	4.1 ± 1.1	4.4 ± 0.8	0.019	0.59	*Streptococcus pyogenes*-lysate2	5.3 ± 1.8	6.2 ± 1.9	0.008	0.20
RhinoA15-Lysate	2.6 ± 0.6	2.9 ± 0.8	0.040	0.76	*Streptococcus pyogenes*-lysate	2.8 ± 0.8	3.2 ± 0.7	0.008	0.20
Astrovirus-Lysate	2.0 ± 0.9	2.1 ± 0.7	0.042	0.76	NoroGII.4-Capsid	9.9 ± 1.0	9.6 ± 1.1	0.027	0.55
NoroGII.4-Capsid	9.4 ± 2.1	9.0 ± 1.9	0.044	0.76					

**Table 3:** List of antigens for which antibody levels are significantly different between ME/CFS and healthy controls by sex. Values are given as log2 MFI averages ± standard deviations. Blue indicates lower antibody levels in cases than in controls. Red indicates lower levels in controls than in cases. *n* = number of antigens with significant differences (*p* < 0.05) in antibody levels between case and control. *p* = *p*-value, q = q-value.

Male (*n* = 42: Top 9 Ranked by *p*-Value + Rotavirus-SA11)					Female (*n* = 4)				
Pathogen	ME/CFS	Control	*p*	q	Pathogen	ME/CFS	Control	*p*	q
*B.burgdorferi*-lysate	1.0 ± 0.6	2.0 ± 0.7	0.000	0.01	*Streptococcus dysgalactiae*-lysate	2.6 ± 0.7	3.2 ± 0.6	0.003	0.39
EBV-EBNA1	3.0 ± 0.9	4.4 ± 1.2	0.002	0.12	*Streptococcus pyogenes*-lysate2	5.2 ± 1.7	6.2 ± 1.9	0.010	0.47
RhinoA15-lysate	2.3 ± 0.5	2.9 ± 0.5	0.005	0.12	*EBV-gp125*	7.1 ± 0.8	6.7 ± 0.9	0.012	0.47
Astrovirus-lysate	1.6 ± 0.3	2.2 ± 0.6	0.005	0.12	Rotavirus-SA11	2.9 ± 0.6	2.6 ± 0.5	0.033	0.98
HEV-Orf2	3.2 ± 0.8	4.1 ± 0.8	0.007	0.12					
RhinoB14-lysate	2.1 ± 0.4	2.5 ± 0.3	0.010	0.12					
SARS2-lysate	1.3 ± 0.4	1.9 ± 0.5	0.010	0.12					
CoxB2-lysate	0.9 ± 0.2	1.4 ± 0.5	0.012	0.12					
*C. trachomatis*-lysate	0.8 ± 0.3	1.3 ± 0.6	0.014	0.12					
Rotavirus-SA11	2.4 ± 0.5	2.9 ± 0.7	0.025	0.12					

## Data Availability

Data supporting the results are available in the Supplemental Data section.

## Supplementary Materials

Supplementary Tables S1–S5 are submitted as separate, individual Excel files. The following supporting information can be downloaded at: https://www.mdpi.com/article/10.3390/proteomes10020021/s1. Table S1: List of Augmenta short names with their respective full pathogen names. Table S2: Comparison of case vs. control median fluorescence intensity antibody levels for all 122 antigens surveyed. Table S3: Comparison of case vs. control median fluorescence intensity antibody levels for all 122 antigens surveyed—outliers removed. Table S4: Complete list of antigens with significantly different antibody levels between ME/CFS and healthy control males. Table S5: Complete list of antigens found to be significantly different between age groups overall (all) and within experimental subgroups (case, control, and female).
